# Resurfacing versus not-resurfacing the patella in one-stage bilateral total knee arthroplasty: a prospective randomized clinical trial

**DOI:** 10.1007/s00264-019-04361-7

**Published:** 2019-06-21

**Authors:** Chengzhi Ha, Baoxin Wang, Wei Li, Kang Sun, Dawei Wang, Qicai Li

**Affiliations:** 1 Department of Joint Surgery, Liaocheng People’s Hospital, Liaocheng, 252000 ShanDong China; 2 Department of Joint Surgery, Affiliated Hospital of Qingdao University, No. 1677 Wutai mountain Road, Economic Development Zone, Qingdao, 266071 Shandong China

**Keywords:** Total knee arthroplasty, Osteoarthritis, Patellar resurfacing, Anterior knee pain

## Abstract

**Purpose:**

Resurfacing the patella in one-stage bilateral total knee arthroplasty (TKA) remains debatable. This study aimed to assess the mid-term outcomes of patients after one-stage bilateral TKA performed with and without patellar resurfacing, respectively, with at least five years of follow-up.

**Methods:**

Sixty-six patients (132 knees) scheduled for first-ever one-stage bilateral TKA due to osteoarthritis received patellar resurfacing and retention, respectively, on one knee and the other, randomly selected. All patients received Scorpio NRG knee prostheses and were evaluated by radiology (anteroposterior, lateral, and axial views) pre-operatively and yearly post-operatively, for at least five years. Knee Society Score and Feller Score values were measured. Anterior knee pain, patellar clunk, and patient satisfaction were assessed.

**Results:**

One patient died within five years of operation and four were lost to follow-up. One patient developed severe dementia and could not be constructively questioned. Therefore, 60 patients (120 knees) were finally analyzed. There were significantly improved Knee Society and Feller scores (*P* < 0.001) in the resurfacing group compared with the non-resurfacing group post-operatively. Anterior knee pain and patellar clunk rates were lower on the resurfaced side compared with the non-resurfaced side (*P* < 0.001). Meanwhile, 47% and only 7% patients preferred the resurfaced and non-resurfaced sides, respectively, at final follow-up. No revision was performed for patellofemoral complications, and no significant differences were found between the two groups in radiographic outcomes.

**Conclusions:**

Using the Scorpio NRG knee prosthesis, patellar resurfacing is superior to non-resurfacing in patients with osteoarthritis observed for ≥ five years.

**Registration trials number:**

NCT03600922

**Key Points:**

• **Findings** Patellar resurfacing is superior to non-resurfacing in osteoarthritis (OA) patients undergoing total knee arthroplasty (TKA) with the Scorpio NRG knee prosthesis.

• **Implications** Patellar resurfacing should be performed in OA patients during TKA.

• **Caution** Several prosthesis types should be assessed in the same study setting, and multicenter studies are required before generalizability of the present findings.

**Electronic supplementary material:**

The online version of this article (10.1007/s00264-019-04361-7) contains supplementary material, which is available to authorized users.

## Introduction

About 11% of all individuals above 64 years of age show symptomatic knee osteoarthritis (KOA) [1]. The most successful operative option for the treatment of advanced KOA is total knee arthroplasty (TKA). The demand for primary TKA is projected to increase to 3.4 million annually by 2030 in the USA [2]. Despite the excellent record for TKA in the treatment of KOA, some patients show poor functional results and persistent anterior knee pain after TKA. This could be attributed to patellofemoral joint problems.

Meanwhile, the optimal treatment of the patellofemoral joint in primary TKA for KOA remains undefined. Viewpoints regarding patellar resurfacing have evolved from no-resurfacing in early 1970s, to systematic resurfacing in the 1980s, and are currently moving toward selective indications [3]. Although there are multiple studies comparing patellar resurfacing and non-resurfacing in TKA [4], the clear superiority of one approach over the other has not been described [5]. Indeed, both options have potential benefits and risks that need to be assessed and balanced based on the surgeon’s experience, preference, and patient’s expectations.

While patellar resurfacing in TKA has been somewhat extensively assessed, only few studies have compared patellar resurfacing and no-resurfacing in one-stage bilateral TKA, in which high peri-operative complications represent a serious concern [6]. In addition, TKA may result in complications such as patellar instability, functional malalignment, anterior knee pain, and patellar clunk syndrome [7]. It is essential to determine whether patellar resurfacing or no-resurfacing in one-stage bilateral TKA is optimal in treating KOA patients.

Therefore, this prospective randomized clinical trial aimed to assess mid-term outcomes of patients after one-stage bilateral TKA performed with and without patellar resurfacing, respectively. The results showed that patellar resurfacing was generally superior to non-resurfacing in patients with KOA observed for at least five years post-operatively.

## Methods

### Study design and patients

This prospective randomized clinical trial assessed patients with KOA undergoing first bilateral TKA in the affiliated hospital of Qingdao University, from March 2011 to August 2012. The inclusion criterion was bilateral knee OA. The exclusion criteria were previous patellectomy, inflammatory arthritis, patellar fracture, patellar instability, previous extensor mechanism procedures, high tibial osteotomy, severe valgus or varus deformity (> 20°), severe flexion contracture (> 30°), previous unicondylar knee replacement, and a history of septic arthritis or osteomyelitis.

The study protocol was approved by the ethical institutional review board of the affiliated hospital of Qingdao University. Signed informed consent was provided by each patient.

### Randomization

A statistical expert blinded to the research procedure generated a random number sequence using a computer. To conceal randomization outcomes, the allocated numbers were placed into concealed opaque envelopes. Randomization was accomplished by opening a randomly selected envelope in the operation room after femoral and tibial cuts, immediately prior to patellar preparation. The left knee received the treatment indicated by the envelope, while the contralateral knee (right knee) received the alternative treatment; either way, the surgery started with the left knee. Thus, all patients had one patella resurfaced and the contralateral patella non-resurfaced.

### Surgery

All patients were operated by a single surgeon (Kang Sun, one of the authors) using Scorpio NRG knee prostheses (Stryker, USA). The tourniquet was tied to the proximal thigh pre-operatively. A midline skin incision and the medial parapatellar approach were used, preserving the infrapatellar fat pad. Tibial bone cuts as well as distal and posterior femoral bone cuts were performed according to mechanical and anatomical axis measurements in each patient. Femoral component rotation was oriented parallel to the transepicondylar axis. The rotation reference for the tibial component was the medial half of the tibial tuberosity. The patellar cut was performed with an oscillating saw on the patellar clamp. Calipers were used to measure the patellar thickness intra-operatively in an attempt to restore the baseline composite height in all resurfacing procedures. Patellar resurfacing was undertaken with a cemented, inset domed component. When resurfacing was not performed, the so-called patelloplasty was carried out, including osteophyte removal and smoothing of fibrillated cartilage. An assessment of patellar tracking was carried out by the no thumb test; if necessary, a lateral retinaculum release was performed. Patellar thicknesses before and after resurfacing were measured.

Post-operative drains were used in all cases and removed on the first post-operative day. Pain control was achieved with epidural patient-controlled analgesia for the first 24 post-operative hours, followed by oral analgesics, as tolerated. The patients were administered antibiotics (cefazolin sodium salt 0.5 g/bid) by intravenous drips for three days to prevent infection. For thromboprophylaxis, all patients were administered subcutaneous low molecular weight heparin (LMWH) at a dose of 4000 AxaIU (0.4 ml)/day, starting 12 hours after the operation. All patients were administered physical rehabilitation therapy by the same rehabilitation technologist, as reported previously [8].

### Follow-up and data collection

The patients were followed post-operatively at three months and annually thereafter. An evaluator not involved in study design but familiar with the assessment tools was responsible for data collection. The evaluator and patients were blinded to the surgical procedure. The primary outcome was the Knee Society System (KSS) score; the system consists of a 100-point scale for clinical status and a 100-point score for function [9].

Secondary outcomes included the Feller score, VAS score for anterior knee pain, patellar clunk and function, and patient satisfaction. Patellar function was evaluated using the Feller score [10]. Anterior knee pain rate as well as patellar clunk and crepitus were assessed. Anterior knee pain was evaluated during a simulated activity of daily living; pain intensity was rated using a visual analog scale (VAS) ranging from 0 to 10 points, with 0 being no pain and 10 representing maximum pain [11]. A score above 5 was defined as anterior knee pain. Patient satisfaction was assessed with questionnaires at each follow-up visit.

Alignment of the TKA components was evaluated by measuring the standard anteroposterior and lateral X-rays of the knee.

All patients received X-ray examination (anteroposterior, lateral, and axial views) before surgery, as well as at three months and one, two, three, four and five years after surgery. For the radiographic assessment of the patellofemoral joint, on the lateral radiograph with the knee in 30° flexion, the Insall-Salvati index was used to calculate the patellar height. The patellar tilt was measured by using the axial view of the knee, measuring the angle between the line tangent to the two femoral condyles or the femoral component, and the line joining the medial and lateral ends of the patella or the line tangent to the base of the patella component. Patellar subluxation was assessed as a percentage compared to the mid-lateral dimension of the femur or femoral component. The radiographs were analyzed by Qicai Li (associate chief physician, one of the authors).

### Statistical analysis

All data analyses were performed with SPSS 19.0 (IBM, Armonk, NY, USA). The sample size was estimated according to a power analysis [12], as follows. A 10-point difference in the 100-point clinical KSS was considered the endpoint of statistical analysis. With an alpha value of 5% and a power of 95%, a sample size of 50 patients (100 knees for both groups) was required. Finally, to compensate for potential loss to follow-up, we recruited a total of 85 patients. Continuous variables were expressed as means ± standard deviation (SD) and compared using the paired *t* test in case of normal distribution confirmed by the Kolmogorov-Smirnov test. The Wilcoxon signed-rank test was used to assess the non-parametric data. The Greenhouse-Geisser test was performed to compare repeated measures data such as KSS and Feller scores. Categorical data, such as anterior knee pain and patellar clunk and crepitus, were compared by the McNemar-Bowker test or the Fisher exact test. *P* < 0.05 was considered statistically significant.

## Results

### Study flowchart

**Fig. 1:**
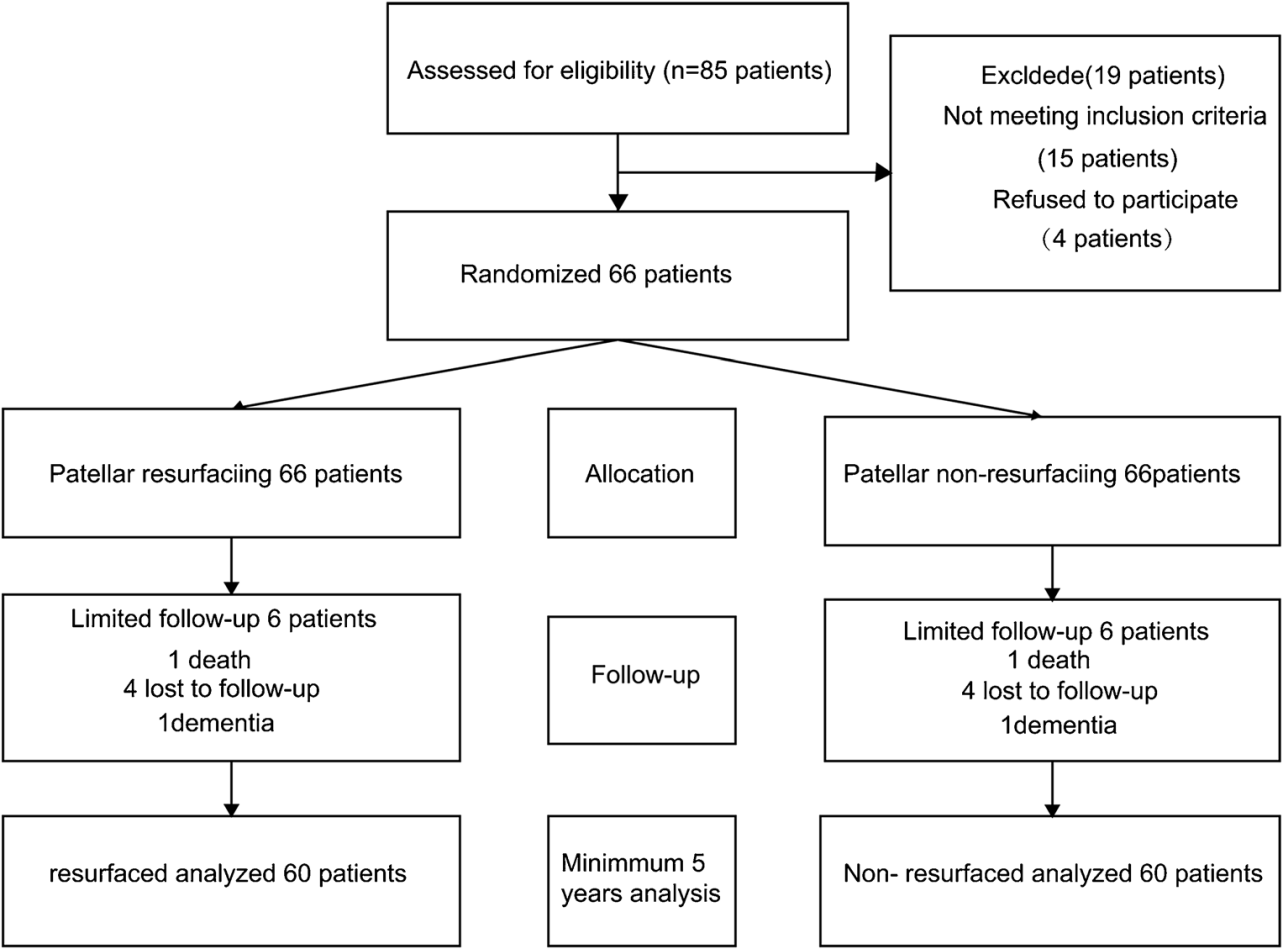
Study flowchart

During the enrollment period, 85 patients with degenerative OA were evaluated for possible inclusion in the study. A total of 15 patients did not meet the inclusion criteria. The 70 patients who met the inclusion criteria were enrolled in the study and 66 patients provided informed consent. One patient died of cerebral haemorrhage within five years of follow-up and four were lost to follow-up. One patient developed severe dementia and was excluded. Finally, 60 patients were included in the final analysis. The study flowchart and the numbers of knees included in the current analysis were outlined in a Consolidated Standards of Reporting Trials (CONSORT) diagram (Fig. 1).

### Patient baseline characteristics

The patients in the study cohort were 65.2 ± 5.4 (range 58–70) years old. There were 38 women and 22 men. BMI was 23.8 ± 4.3 (range 18.9–28.2) kg/m^2^. The patients’ baseline characteristics are summarized in Table 1.

**Table 1:** Demographic characteristics

	Mean ± SD/*n* (%)	Range
*N* = 60		
Age (years)	65.2 ± 5.4	58–70
Gender, *n* (%)		
Male	38 (63.3)	
Female	22 (36.7)	
BMI (kg/m^2^)	23.8 ± 4.3	18.9–28.2
Follow-up (months)	66.4 ± 3.2	61–78

### Surgical outcomes

The lateral retinaculum was released in no patients. The patellar resurfacing procedure affected the operative time; compared with the group without patellar replacement, TKA operation with patellar replacement showed an operation 3.48-min longer (*P* < 0.001). Mean peri-operative blood loss was 36.18 ± 3.46 ml in the patellar resurfacing group and 36.20 ± 3.39 ml in the non-resurfacing group, indicating no statistically significant difference between the two groups (*P* = 0.957). Post-operatively, mean patellar thicknesses were 21.08 ± 1.39 mm and 22.03 ± 1.68 mm in the patellar resurfacing and non-resurfacing groups, respectively. The mean difference of 0.95 mm was statistically significant (*P* < 0.001) (Table 2).

**Table 2:** Pre-operative and intra-operative features

	PR group *N* = 60	N-PR group *N* = 60	*P* value
Pre-operative Kellgren-Lawrence classification			0.803
III	10	9	
IV	50	51	
Operation side			0.465
Left	32	28	
Right	28	32	
Operative time (minutes)	41.70 ± 2.55	38.22 ± 2.70	< 0.001
Blood loss (ml)	36.18 ± 3.46	36.20 ± 3.39	0.957
Post-operative patellar thickness (mm)	21.08 ± 1.39	22.03 ± 1.68	< 0.001

*PR*, patellar resurfacing; *NP*, non-patellar resurfacing

### KSS scores

There were no significant differences in pre-operative KSS (clinical and function) scores between the resurfacing and non-resurfacing groups, as shown in Table 3 (*P* = 0.31 and *P* = 0.15, respectively). Both groups showed significant improvements in both KSS scores after surgery; the time effect was significant (both *P* < 0.001). There were significantly better scores for the resurfaced sides compared with the non-resurfaced side at annual follow-up; the main effect of group was significant (both *P* < 0.001).

**Table 3:** KSS clinical and function scores in the resurfacing and non-resurfacing group

		Pre-operation	3 months after operation	1 year after operation	2 years after operation	3 years after operation	4 years after operation	5 years after operation
Clinical scores	PR (*N* = 60)	45.73 ± 3.05	68.80 ± 3.13	79.12 ± 3.22	87.32 ± 2.52	89.70 ± 1.81	90.80 ± 1.59	92.07 ± 1.45
	N-PR (*N* = 60)	45.38 ± 3.14	67.75 ± 2.58	78.08 ± 3.41	86.28 ± 2.99	88.83 ± 2.64	89.62 ± 2.22	90.98 ± 2.33
	*P* value	*t* = 1.03 *P* = 0.31	Time effect *F* = 5243.14, *P* < 0.001 Main effect *F* = 17.57, *P* < 0.001					
Function scores	PR *(N* = 60)	38.65 ± 4.47	47.65 ± 3.65	59.73 ± 4.89	64.33 ± 2.94	69.87 ± 3.64	78.02 ± 3.19	80.37 ± 3.02
	N-PR (*N* = 60)	39.22 ± 4.04	46.63 ± 3.92	58.13 ± 4.29	62.83 ± 3.55	68.17 ± 2.85	75.78 ± 3.82	78.10 ± 3.23
	*P* value	*t* = − 1.46 *P* = 0.15	Time effect *F* = 2238.28, *P* < 0.001 Main effect *F* = 15.81, *P* < 0.001					

*KSS*, Knee Society Scores; *PR*, patellar resurfacing; *N-PR*, non-patellar resurfacing

### Feller scores

There was no significant difference in pre-operative Feller scores between the two groups as shown in Supplementary Table 2 (*P* = 0.959). Both treatment groups had significant improvement in Feller scores post-operation; the time effect was significant (time effect *P* < 0.001). Feller scores were obviously higher in the patellar resurfacing group compared with the non-patellar resurfacing group at three months and at one, two, three, four and five years after surgery. The differences were statistically significant, indicating that patellofemoral joint function in the resurfacing group was better than that of the non-resurfacing group (main effect *P* < 0.001) (Supplementary Table 2).

### Anterior knee pain rates

Persistent anterior knee pain (AKP) was noted in 14 patients (23%) on the non-resurfaced side, whereas three (5%) patients reported such pain on the resurfaced side within three months of surgery. With time, the rate of AKP gradually decreased in both groups. One patient had similar pain intensity in the bilateral knee at the last follow-up. No patients with persistent pain showed symptoms severe enough to require further surgery **(**Supplementary Table 3).

### Patellar clunk syndrome rates

At the final follow-up, six (10%) and 24 (40%) patients showed patellar clunk syndrome in the patellar resurfacing and non-patellar resurfacing groups, respectively, indicating a statistically significant difference (*P* < 0.001) (Supplementary Table 3). Two patients suffered from bilateral patellar clunk syndrome and had severe valgus alignment pre-operatively, although the mechanical axis was corrected post-operatively. In addition, post-operative patellar thickness in one patient with the patellar clunk syndrome in the patellar resurfacing group was greater than pre-operative patellar thickness (1 mm). Patellar component size in three patients with the patellar clunk syndrome in the patellar resurfacing group was below 38 mm.

### Radiographic findings

Pre-operatively, the mean pre-operative mechanical axis was − 5.65° ± 1.02° (varus) on the patellar resurfacing side, compared to − 5.45° ± 0.98° (varus) on the non-patellar resurfacing side (*P* = 0.71). Five knees had valgus alignment > 10°, including 3 and 2 in the non-resurfaced and resurfaced groups, respectively. Post-operatively, the mechanical axis in all patients was corrected to 5.82° ± 1.13° (valgus) on the patellar resurfacing side and 5.78° ± 1.04° (valgus) on the non-patellar resurfacing side (*P* = 0.91). Post-operatively, alignment of the femoral and tibial prosthesis components showed no significant difference between the two groups (*P* = 0.91). Post-operative patellar tracking was considered to be clinically satisfactory in all cases. Mean patellar tilt was greater in knees with patellar resurfacing compared with the non-resurfacing group, but the difference was not significant (*P* = 0.68). The Insall-Salvati index in the two groups showed no significant difference after TKA (*P* = 0.93). There was no change in joint line position by > 5 mm in either group (Supplementary Table 4).

### Complications

No patients at the last follow-up had required revision for patellofemoral problems. There were no cases of patellar subluxation or dislocation, rupture of the quadriceps tendon, aseptic component loosening, patellar osteonecrosis, patellar fragmentation, or periprosthetic fracture.

### Patient satisfaction

Regarding subjective preference, 12 (20%) patients affirmed to prefer the resurfaced side and six (10%) preferred the non-resurfaced counterpart, while 42 (70%) expressed no preference at three months after operation. However, with follow-up time, more and more patients preferred the resurfaced side (47%) and only 7% patients preferred the non-resurfaced side at the final follow-up.

### Representative case

**Fig. 2:**
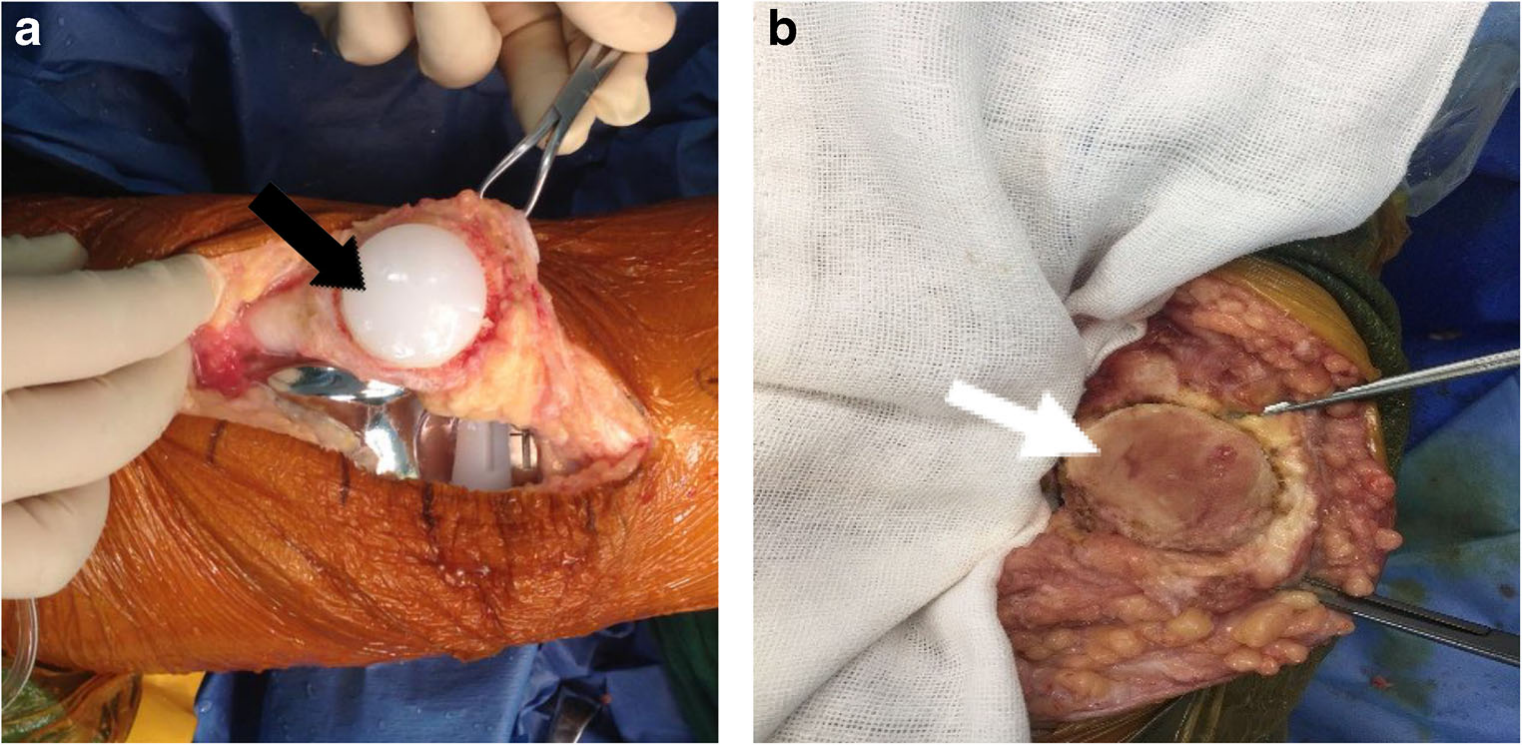
A 68-year-old woman with a preoperative diagnosis of bilateral osteoarthritis of the knee joint. **a** Left: resurfaced patella (black arrow). **b** Right: non-resurfaced patella (white arrow)

**Fig. 3:**
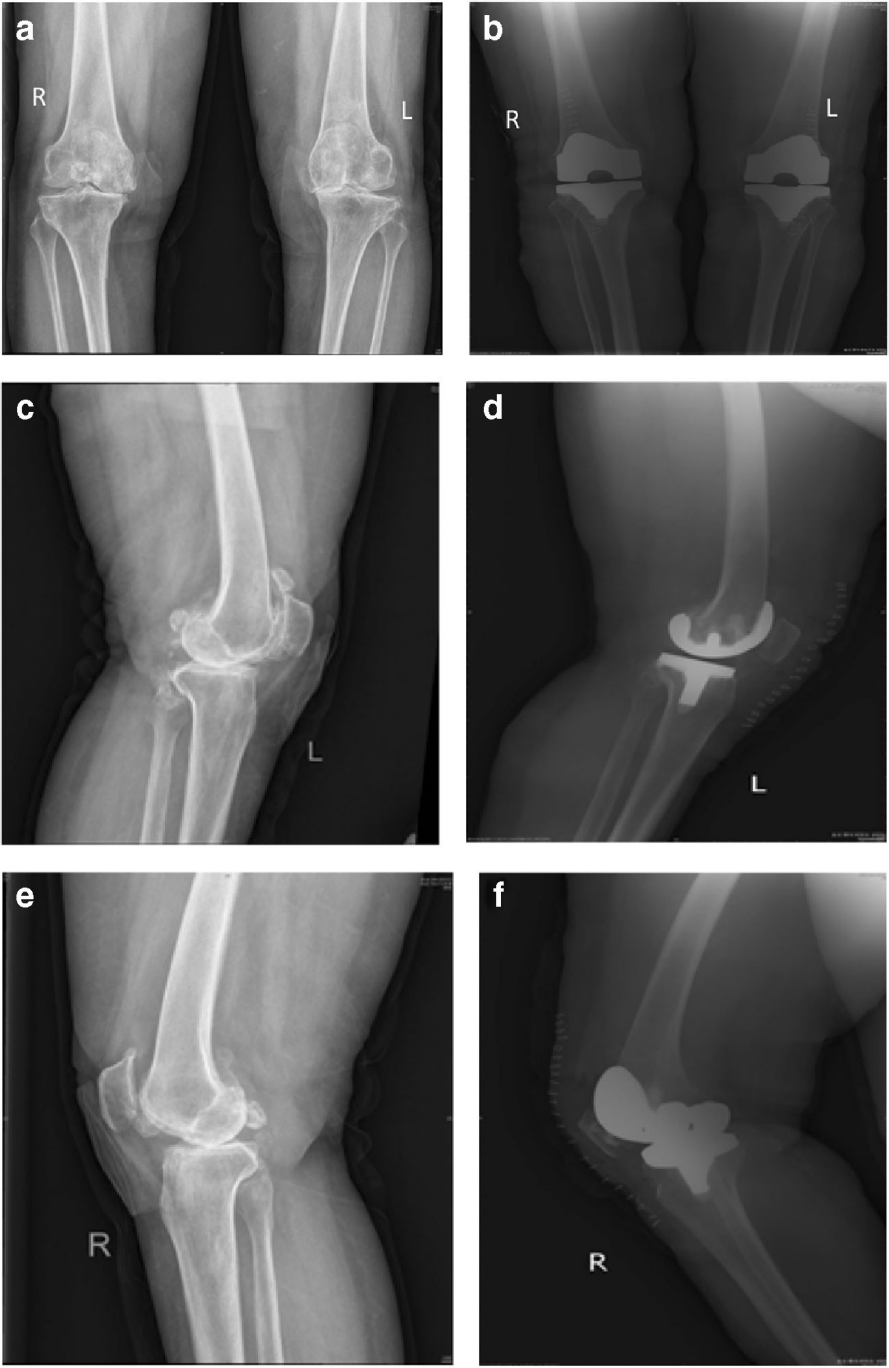
Pre-operative (1 week) and post-operative (3 days) X-ray examinations of a 68-year-old woman with bilateral osteoarthritis. Left: non-resurfaced patella. Right: resurfaced patella. **a** Anteroposterior position 1 week before operation. **b** Anteroposterior position 3 days after operation. **c** Lateral position of left knee joint 1 week before operation **d** Lateral position of left knee joint 3 days after operation (non-resurfaced). **e** Lateral position of right knee joint 1 week before operation. **f** Lateral position of right knee joint 3 days after operation (resurfaced)

**Fig. 4:**
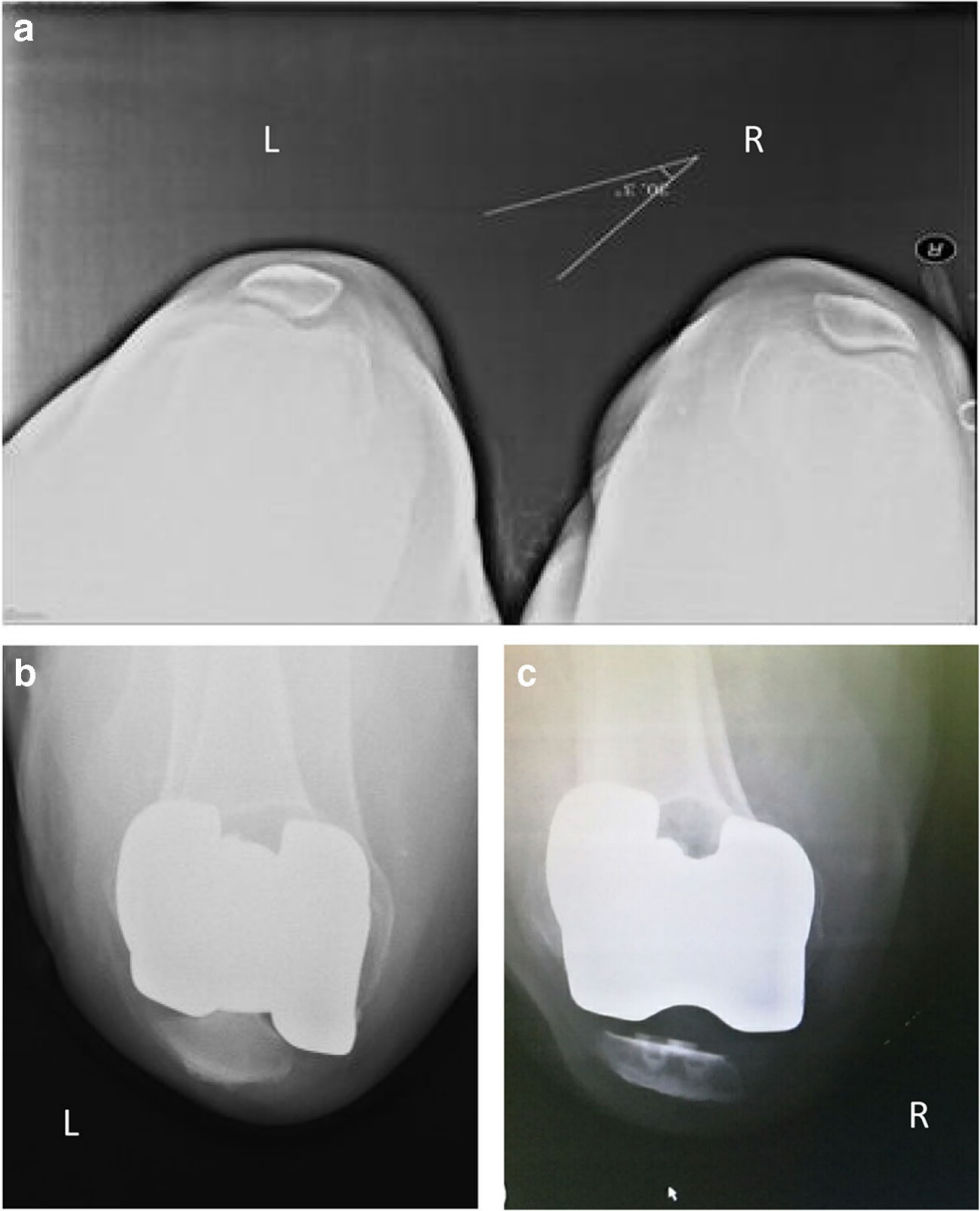
Pre-operative (3 days) and post-operative (1 month) X-ray examinations (axial patella view) of a 68-year-old woman with bilateral osteoarthritis. Left: non-resurfaced patella. Right: resurfaced patella. One-month after the operation, the patient’s pain was significantly relieved, the activities were recovered well, and the post-operative joint flexion activity reached 110°. **a** Axial position 3 days before operation. **b** Left knee joint in the axial position 1 month after operation. **c** Right knee joint in axial position 1 month after operation

Figures 2, 3, and 4 present the case of a woman of 68 years of age with bilateral KOA.

## Discussion

This work strongly suggests that TKA relieved pain and improved function in patients with KOA with or without patellar resurfacing, as previously reported [13]. Nevertheless, we found significantly improved KSS and Feller scores for the resurfaced side compared with the non-resurfaced side at annual follow-up visits. Other advantages of resurfacing the patella during TKA included reduced incidence rates of AKP and patellar clunk syndrome.

Although previous findings reported no significant difference in KSS scores between patients with resurfaced or non-resurfaced patella [14], the KSS and Feller scores in the patellar resurfacing group were significantly higher than those of the non-patellar resurfacing group after three months and one, two, three, four and five years. These findings indicated that the patellofemoral joint function is more pronouncedly improved in the resurfacing side compared with the non-resurfacing side. These advantages mainly involve walking and stair climbing. Our results were consistent with Kordelle et al. [15].

Persistent AKP remains an important clinical issue after TKA. Its exact aetiology remains elusive, and the effects of prosthesis design, surgical technique, the degree of patellar chondromalacia, pre-operative AKP, and patellar tracking alteration on the prevalence of post-operative AKP remain undefined [16]. A previous study reported an average AKP incidence in non-resurfaced patients of 10%, versus 3.3% for resurfaced cases [17], corroborating our findings. At five years of follow-up, there were 5% patients with persistent AKP on the resurfaced side, versus 23% cases complaining of such pain in the non-resurfaced side. The overall incidence of AKP was higher in the current study compared with previous reports. The use of different scoring systems has resulted in variations in objective AKP assessment, contributing to the observed heterogeneity.

The term “patellar clunk syndrome” was first introduced by Hozack in 1989 [18]. In this study, at five years of follow-up, the rate of patellar clunk syndrome was obviously lower in the patellar resurfacing side compared with the patellar non-resurfacing side. The surgical technique, patellar shape, abnormal patellar tracking, soft tissue imbalance, femoral component design, and positioning have been implicated in the aetiology of the patellar clunk syndrome [19]. We speculate that abnormal patellar tracking could be one of the causes of such excessive peripatellar fibrosis.

Prosthesis design is another cause of patellar clunk syndrome [20], whose incidence ranges from 0 to 25% for different knee prostheses [21]. A previous study found that femoral prostheses with a deepened trochlear groove, posterior intercondylar box, and smooth box transition appear to reduce patellar clunk syndrome occurrence [22]. This may prevent suprapatellar nodule formation by decreasing the impingement of distal quadriceps tendon on the anterosuperior edge of the intercondylar box [23]. The intercondylar box of the Scorpio NRG knee prosthesis is likely somewhat more frontal compared with other posterior-stabilized prostheses. This may be why the Scorpio NRG knee prosthesis causes the patella clunk syndrome.

Roessler et al. [24] showed that patellar tilt, width, and thickness, as well as tibial component positioning, could be predictive for the need for secondary patellar resurfacing. Franck et al. [25] showed that patellofemoral dysplasia, KOA, and maltracking should be detected pre-operatively and would indicate the need for resurfacing during TKA. Prudhon et al. [26] showed that the patella should be assessed in the sagittal, frontal, and horizontal planes to determine adequately the patella height and determine the risk of secondary TKA. After secondary resurfacing of the patella, better outcomes were observed in patients without patellar tilting [27]. A Norwegian study showed that primary resurfacing had better outcomes than secondary resurfacing [28]. Nevertheless, another study showed that secondary patellar resurfacing led to good patient satisfaction [27]. In the present study, the resurfacing itself was associated with better outcomes.

The main limitation of this study is that it was performed in a single centre, and other institutions should carry out similar investigations to confirm the present results. In the present study, bilateral anterior knee pain was not an exclusion criterion, which was indeed a limitation for this study that could bias pain scoring. There are many scoring systems for knee joints, but none of them is objective and complete. In addition, it would be useful to assess several prosthesis types in the same study setting, for a comprehensive comparison between the two methods.

## Conclusion

In conclusion, our results showed that with the Scorpio NRG knee prosthesis, patellar resurfacing is a better option compared with non-resurfacing for at least five years in OA patients.

## Electronic supplementary material

Supplementary Table 1 (DOCX 21 kb).

Supplementary Table 2 (DOCX 22 kb).

Supplementary Table 3 (DOCX 21 kb).

Supplementary Table 4 (DOCX 22 kb).
