# Supplementary material for: Resurfacing versus not-resurfacing the patella in one-stage bilateral total knee arthroplasty: a prospective randomized clinical trial
Source: Int Orthop. 2019 Jun 21;43(11):2519–27. doi: 10.1007/s00264-019-04361-7 (PMC6848038; doi:10.1007/s00264-019-04361-7)
Supplement: Supplementary file 1 — (DOCX 21 kb). [file 264_2019_4361_MOESM1_ESM.docx]

**Supplementary Table 1**. Patellar score details

|  | Score |
| --- | --- |
| Anterior knee pain |  |
| None | 15 |
| Mild | 10 |
| Moderate | 5 |
| Severe | 0 |
| Quadriceps strength |  |
| Good (5/5) | 5 |
| Fair (4/5) | 3 |
| Poor (</5) | 1 |
| Ability to rise from chair |  |
| Able with ease (no arms) | 5 |
| Able with ease (with arms) | 3 |
| Able with difficulty | 1 |
| Unable | 0 |
| Stair-climbing |  |
| 1 foot/stair, no support | 5 |
| 1 foot/stair, with support | 4 |
| 2 feet/stair, no support | 3 |
| 2 feet/stair, with support | 2 |
