# Supplementary material for: Resurfacing versus not-resurfacing the patella in one-stage bilateral total knee arthroplasty: a prospective randomized clinical trial
Source: Int Orthop. 2019 Jun 21;43(11):2519–27. doi: 10.1007/s00264-019-04361-7 (PMC6848038; doi:10.1007/s00264-019-04361-7)
Supplement: Supplementary file 2 — (DOCX 22 kb). [file 264_2019_4361_MOESM2_ESM.docx]

**Supplementary Table 2.** Feller scores in the resurfacing and non-resurfacing groups

|  | Pre-operation | 3 months after operation | 1 year after operation | 2 years after operation | 3 years after operation | 4 years after operation | 5 years after operation |
| --- | --- | --- | --- | --- | --- | --- | --- |
| PR (N=60) | 17.43±2.27 | 19.55±2.37 | 21.67±2.38 | 22.68±2.22 | 22.77±2.09 | 23.35±2.23 | 23.87±2.05 |
| N-PR (N=60) | 17.42±1.81 | 18.32±2.47 | 19.77±2.53 | 20.80±2.70 | 21.03±2.73 | 21.55±2.98 | 21.70±3.10 |
| *P* value | t=0.051  *P*=0.959 | Time effect F=193.92, *P* <0.001  Main effect F=18.06, *P* <0.001 | | | | | |

PR, patellar resurfacing N-PR, non-patellar resurfacing
