# Supplementary material for: Resurfacing versus not-resurfacing the patella in one-stage bilateral total knee arthroplasty: a prospective randomized clinical trial
Source: Int Orthop. 2019 Jun 21;43(11):2519–27. doi: 10.1007/s00264-019-04361-7 (PMC6848038; doi:10.1007/s00264-019-04361-7)
Supplement: Supplementary file 3 — (DOCX 21 kb). [file 264_2019_4361_MOESM3_ESM.docx]

**Supplementary Table 3** Patellofemoral characteristics in the resurfacing and non-resurfacing groups

| No. of knees (%) | PR  N=60 | N-PR  N=60 | *P* value |
| --- | --- | --- | --- |
| Anterior knee pain | 3 (5%) | 14 (23%) | <0.001 |
| Clunk of patella | 6 (10%) | 24 (40%) | <0.001 |

PR, patellar resurfacing N-PR, non-patellar resurfacing
