# Supplementary material for: Resurfacing versus not-resurfacing the patella in one-stage bilateral total knee arthroplasty: a prospective randomized clinical trial
Source: Int Orthop. 2019 Jun 21;43(11):2519–27. doi: 10.1007/s00264-019-04361-7 (PMC6848038; doi:10.1007/s00264-019-04361-7)
Supplement: Supplementary file 4 — (DOCX 22 kb). [file 264_2019_4361_MOESM4_ESM.docx]

**Supplementary Table 4**. Radiographic findings

|  | PR  N=60 | N-PR  N=60 | *P* value |
| --- | --- | --- | --- |
| Preoperative alignment | Varus -5.65°±1.02° | Varus -5.45°±0.98° | 0.71 |
| Postoperative alignment | Valgus 5.82°±1.13° | Valgus 5.78°±1.04° | 0.91 |
| Preoperative Insall-Salvati index | 1.05 ± 0.18 | 1.04 ± 0.16 | 0.92 |
| Postoperative Insall-Salvati index | 1.01 ± 0.09 | 1.02 ± 0.07 | 0.93 |
| Preoperative patellar tilt angle | 6.03°± 1.12 | 5.98°± 1.24 | 0.87 |
| Postoperative patellar tilt angle | 4.98°± 1.08 | 4.45°± 1.12 | 0.68 |
| Change in joint line in reference tibial tuberosity (mm) | 2.41 ± 1.22 | 2.76 ± 1.65 | 0.72 |

PR, patellar resurfacing; N-PR, non-patellar resurfacing
